# Redefining light-chain smoldering multiple myeloma: prevalence and progression risk

**DOI:** 10.1038/s41375-026-03028-8

**Published:** 2026-07-06

**Authors:** Cecilie Velsoe Maeng, Sigrún Thorsteinsdóttir, Jón Þórir Óskarsson, Sæmundur Rögnvaldsson, Thórir Einarsson Long, Christian Brieghel, Morten Nørgaard Andersen, Andri Ólafsson, Ásdís R. Þórðardóttir, Brynjar Viðarsson, Páll T. Önundarson, Bjarni A. Agnarsson, Margrét Sigurðardóttir, Ingunn Þorsteinsdóttir, Ísleifur Ólafsson, Elías Eyþórsson, Ásbjörn Jónsson, Stephen Harding, Ola Landgren, Thorvardur Jon Love, Kirsten Grønbæk, Carsten U. Niemann, Sigurður Y. Kristinsson

**Affiliations:** 1 Department of Hematology, Copenhagen University Hospital (Rigshospitalet), Copenhagen, Denmark; 2 Biotech Research and Innovation Center (BRIC), University of Copenhagen, Copenhagen, Denmark; 3 Faculty of Medicine, University of Iceland, Reykjavík, Iceland; 4 Landspítali University Hospital, Reykjavík, Iceland; 5 Department of Medicine, Sahlgrenska University Hospital, Gothenburg, Sweden; 6 Skåne University Hospital, Lund, Sweden; 7 Danish Cancer Institute, Copenhagen, Denmark; 8 Department of Hematology, Aarhus University Hospital, Aarhus, Denmark; 9 Department of Clinical Medicine, Aarhus University, Aarhus, Denmark; 10 Akureyri Hospital, Akureyri, Iceland; 11 The Binding Site- Thermo Fischer Scientific, Birmingham, West Midlands UK; 12 Sylvester Comprehensive Cancer Center, University of Miami, Miami, FL USA; 13 Department of Clinical Medicine, Faculty of Health and Medical Sciences, University of Copenhagen, Copenhagen, Denmark

**Keywords:** Cancer epidemiology, Epidemiology

## Introduction

Smoldering multiple myeloma (SMM) is a precursor to multiple myeloma (MM) and present in 0.5% of the population over 40 years old [1, 2]. In 10–20% of MM patients, the clonal BMPC do not produce intact M proteins but only free light chains (FLC) [3, 4]. This subgroup termed light-chain MM and well-characterized, but light-chain SMM is not clearly defined.

Historically, monoclonal FLC were detected by urine protein electrophoresis (UPEP), but methods of detecting FLC in serum have become widely available and are used to infer the presence of monoclonal FLC. The higher sensitivity of serum FLC analysis and the convenience of a single blood sample over a 24-h urine collection have made this the preferred method for evaluating monoclonal gammopathies in clinical practice [5–7].

The only prior study on light-chain SMM, a single-center retrospective analysis, defined the diagnosis based on urinary monoclonal FLC (≥0.5 g/24 h) and/or >10% BMPC, in the absence of intact serum M protein and end-organ damage [8]. However, this study lacked FLC measurements in many cases, and bone marrow evaluation was missing in up to 40%. In addition, some individuals (27 of 73 tested) would now meet criteria for MM due to an FLC ratio >100. Thus, a refined diagnostic criteria and characterization of light-chain SMM is needed.

The aim of this study was to define and characterize light-chain SMM using FLC analysis and bone marrow sampling within the Iceland Screens, Treats, or Prevents Multiple Myeloma (iStopMM) study. In addition, to externally validate the proposed definition in a population-based clinical cohort in Denmark (the Danish Lymphoid-lineage Cancer Research; DALY-CARE) and to estimate the progression risk.

## Methods

### iStopMM

The iStopMM study is a nationwide screening study [9], summarized in Supplementary Methods. For this study, we defined light-chain SMM by an abnormal FLC ratio and elevation of the involved FLC according to the iStopMM revised FLC reference ranges [10, 11], a BMPC 10–59% and absence of M protein and myeloma defining events [5]. The Mayo Clinic 2/20/20 risk stratification model was used to stratify patients into risk groups. Mass spectrometry (MS) on blood samples and next-generation flow (NGF) on bone marrow samples was performed (elaborated in Supplementary Methods).

Estimates of the proportion of light-chain SMM among individuals with abnormal FLC levels, as well as prevalence calculations for light-chain SMM, were based on individuals randomized to bone marrow sampling and CT scan regardless of laboratory results (Study Arm 3, Supplementary Methods). The point prevalence was determined with a fitted binomial function corrected for age and sex. The standard errors of the fit were used to calculate 95% confidence intervals (CIs) of the prevalence.

### DALY-CARE

The DALY-CARE data resource [12] is a nationwide collection of Danish registries and databases. The SMM cohort was defined as individuals with an ICD-10 coded MM diagnosis who had not died or received anti-myeloma treatment within 90 days of diagnosis. Pathology reports were evaluated to confirm BMPC infiltration of 10–59% in bone marrow biopsy within 90 days of diagnosis. Light-chain SMM was defined as SMM individuals without a detected intact heavy-chain M protein and abnormal values and ratio of FLC at diagnosis. Further details on data and cohort construction in Supplementary Methods and Supplementary Fig. 1.

Progression was defined as start of anti-myeloma treatment or diagnosis of amyloid light-chain (AL) amyloidosis. Individuals were followed from diagnosis to progression, death, or end of follow-up. The median follow-up was calculated using reversed Kaplan–Meier. Overall cumulative incidences were calculated using Aalen-Johansen estimation, accounting for death as a competing risk, and Kaplan–Meier estimation, when not accounting for death as competing risk. Average annual progression rate was calculated as the number of observed progressions per person-year.

Individuals were stratified into 2/20/20 risk groups and cumulative incidence of progression were estimated for each strata. Gray´s test was used to test differences of cumulative incidence curves.

## Results

### iStopMM

A total of 75,422 individuals underwent screening between 2016 and 2020. Of these, 3788 individuals had an abnormal screening test and were randomized to one of the three study arms. A total of 193 participants had SMM based on initial evaluation (Supplementary Fig. 1), of which 23 fulfilled our suggested criteria for light-chain SMM. The cohort characteristics and laboratory results are summarized in Table 1. In addition to the MS results shown in Table 1, IgM peaks below the detection limit of SPEP (0.0015–0.005 g/dl in size) without an associated light chain were identified in 8 individuals (40%).

**Table 1:** Baseline characteristics of individuals with light chain smoldering multiple myeloma.

Variable	Light-chain SMM in iStopMM (*n* = 23)	Light-chain SMM in DALY-CARE (*n* = 88)
**Age, years**	69 (56–77)	73 (66–80)
**Sex**		
Male	16 (70)	53 (60)
Female	7 (30)	35 (40)
**Laboratory values**		
Hemoglobin, g/dL	14.3 (13.1–14.5)	13.2 (11.8–14.0)
Creatinine, µmol/L	85 (73–92.5)	80 (66–107)
Calcium, mmol/L	2.4 (2.34–2.39)	2.4 (2.3–2.5)
Free kappa, mg/L	52.4 (10.2–232.2)	200 (20–441)
Free lambda, mg/L	14 (10.2–154.0)	16 (11–251)
Involved/uninvolved FLC ratio >20	11 (47)	56 (64)
**Immunofixation**		
Negative	13 (57)	61 (76)
Kappa	4 (17)	4 (5)
Lambda	6 (26)	15 (19)
Missing		8
**Isotype (according to FLC analysis)**		
Kappa	15 (65)	60 (68)
Lambda	8 (35)	28 (32)
**24-h UPEP and immunofixation results**		
Detectable	13 (72)	31 (63)
Not detectable	5 (28)	18 (37)
Missing	5	39
**Isotype of urinary light-chain, if detectable**		
Kappa	10 (55)	17 (54)
Lambda	3 (17)	13 (46)
Missing	0	1
**Quantification of urinary light-chain, if detectable**		
Median g/24 h	0.18 g (<0.10–0.61)	0.02 g (0.02–0.26)
>0.2 g/24 h	4 (22)	6 (26)
>0.5 g/24 h	2 (11)	3 (13)
Missing	5	8
**Mass spectrometry results**	20 (87)	
Kappa peak	11 (55)	
Lambda peak	8 (40)	Not available
IgG (0.029 g/dL) and a kappa peak	1 (5)	
IgA (<0.005 g/dL)	1 (5)	
**Bone marrow plasma cells**		
10–19%	20 (87)	56 (64)
20–59%	3 (13)	32 (36)
**Next generation flow results**	19 (83)	
Detectable plasma cell clone in BM	19 (100)	Not available
>95% aberrant plasma cells	6 (32)	
**Number of suppressed immunoglobulins**		
1	3 (13)	23 (27)
2-3	6 (26)	37 (44)
Immunoglobulins >25% below normal range	3 (13)	44 (52)
Data not available	1	3
**Risk group by 2/20/20 risk mode**l		
Low risk, no. (%)	11 (48)	23 (26)
Intermediate risk, no. (%)	10 (43)	44 (48)
High risk, no. (%)	2 (9)	21 (23)

Data is presented as median (interquartal range) for continuous variables and number (%) for categorical variables.

*BM* bone marrow, *FLC* free light chain, *IQR* interquartal range, *SMM* smoldering multiple myeloma, *MM* multiple myeloma, *PB* peripheral blood, *UPEP* urine protein electrophoresis.

AL amyloidosis was screened for with N-terminal pro B-type natriuretic peptide and troponin T measurements in 19 (83%) patients and Congo red staining of bone marrow biopsies in 14 (61%) individuals. No cases of AL amyloidosis were diagnosed.

A total of 1303 individuals were randomized to bone marrow examination and CT scan regardless of SPEP and FLC results. Of these, 105 (8%) had an abnormal FLC analysis and no detectable intact monoclonal immunoglobulins on SPEP. Of those, 86 underwent bone marrow sampling, of whom 16 (19%) had >10% BMPC fulfilling our suggested criteria for light-chain SMM. A total of 112 (11% of those with bone marrow sampling performed) individuals had >10% BMPC and no myeloma defining events. The proportion of light-chain SMM of all SMM in this sub-cohort was therefore 14% (16/112). The prevalence of light-chain SMM in individuals over 40 years old was estimated to be 0.08% (95% CI: 0.07–0.10%), 0.07% (95% CI: 0.05–0.10%) in women, and 0.09% (95% CI: 0.07–0.12%) in men.

### The DALY-CARE cohort

**Fig. 1:**
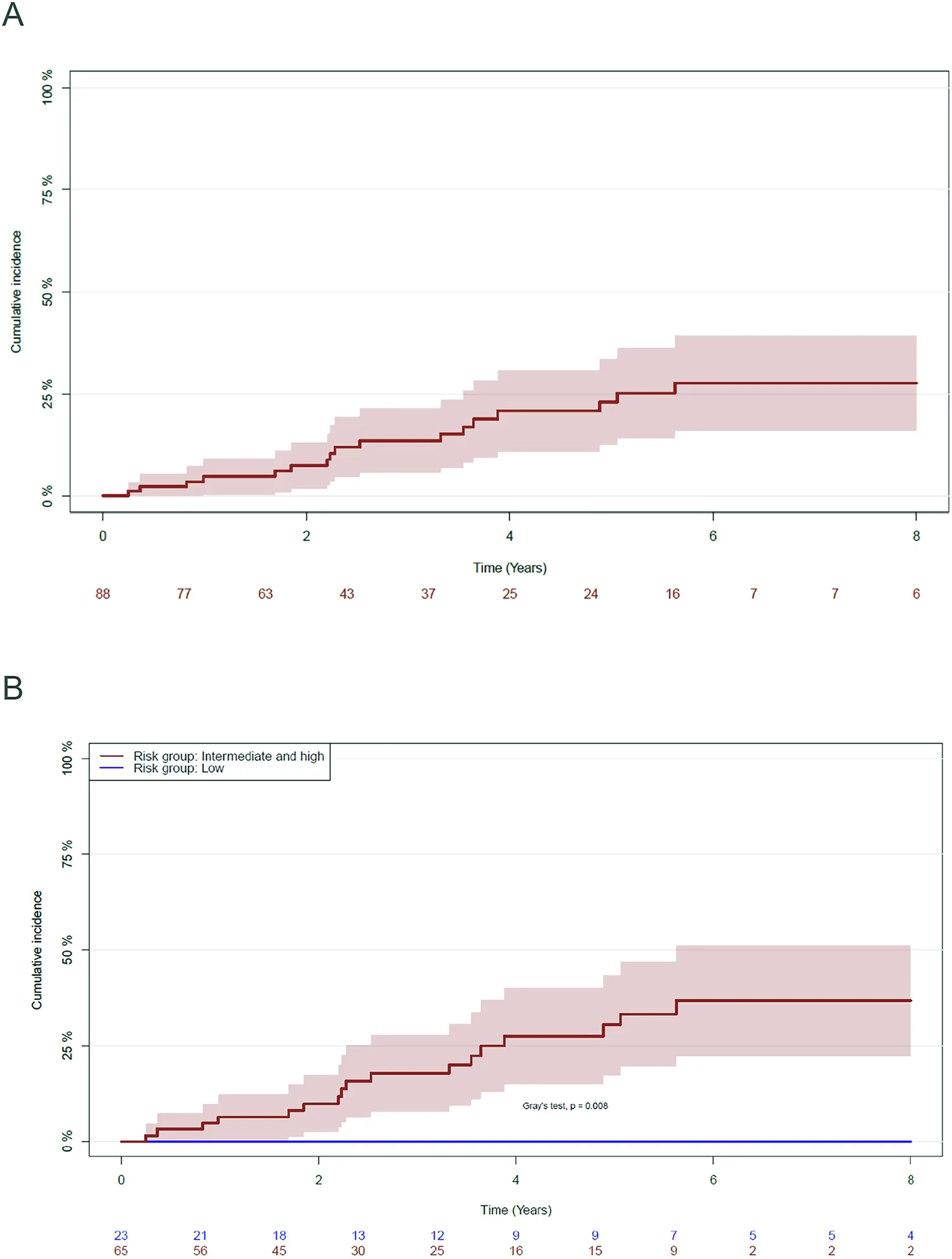
Cumulative incidence of progression in light-chain smoldering multiple myeloma with death as competing risk. **A** Cumulative progression to multiple myeloma or amyloid light-chain  amyloidosis over time in 88 individuals in the DALY-CARE cohort. **B** Cumulative progression to multiple myeloma or amyloid light-chain amyloidosis over time by risk group according to the 2/20/20 risk stratification criteria.

A total of 88 patients in the Danish cohort fulfilled the set criteria for light-chain SMM. The cohort characteristics and laboratory results at diagnosis are summarized in Table 1. The median follow-up was 5.2 years (IQR 2.2–8.3 years). During follow-up, 17 (19%) patients progressed (16 to MM and one to AL amyloidosis), and 30 (34%) died without progression. The estimated cumulative incidence of progression was 8.1 (95% CI: 1.6–14.2%) two years from light-chain SMM diagnosis and 29.5% (95% CI: 14.7–41.8%) at five years. When death was considered a competing risk, the cumulative incidence was 7.4% (95% CI: 1.7–13.2%) two years from light-chain SMM diagnosis and 23.0% (95% CI: 12.5–33.5%) (Fig. 1A). The annualized average progression rate was 6.0% (95% CI 3.5–9.6) (17 progressions during 282 person-years).

Of the 88 individuals with available data for risk stratification according to the 2/20/20 model, 23 (26%) classified as low-risk and 65 (74%) as intermediate- or high-risk SMM. For the low-risk group, no progressions occurred during follow-up. For the intermediate/high-risk individuals, the cumulative incidence was 9.8% (95% CI 2.4–17.3) at two years and 30.3% (95% CI 17.1–43.5) at five years considering death as competing risk (Fig. 1B). The annual rate of progression was 9.2% (95% CI 5.4–14.7) for intermediate/high risk individuals. Of the 16 individuals who progressed to MM, anemia was present in 10 (63%) and renal insufficiency in 8 (50%) at progression. FLC ratio >100 was observed in 10 (63%), of whom 9 also met at least one additional MM diagnostic criterion. For cases with available clinical treatment indications from EHR (*n* = 8), anemia, renal impairment, or bone disease led to treatment initiation in five cases, and rapidly rising FLC levels or FLC ratio >100 in three.

## Discussion

We propose to revise the definition of light-chain SMM to abnormal serum FLC values, absence of detectable intact immunoglobulin on SPEP and immunofixation, and BMPC > 10% without myeloma-defining events. From nationwide screening and systematic bone marrow sampling, we determined that 19% of individuals with abnormal FLC levels had light-chain SMM, corresponding to a population prevalence of 0.08% among individuals over 40 years.

This definition reflects current practice, where serum FLC testing has increasingly replaced UPEP for detecting and monitoring monoclonal light-chain disorders. UPEP was negative in 28% of light-chain SMM cases with available data in iStopMM and 37% in DALY-CARE, suggesting that FLC analysis may be more sensitive for detection. Although Kyle et al. [8] found that 12% of individuals with a detectable Bence Jones proteinuria had a normal FLC ratio (0.26–1.65), indicating that UPEP can be more sensitive in some cases, this requires reassessment using revised FLC reference intervals [10, 11]. While 24-h urine testing may contribute to risk stratification, it is burdensome and may not be necessary at initial evaluation if serum FLC testing is available.

In DALY-CARE, light-chain SMM was associated with an annual progression risk of ~6%, which is similar to previously reported progression risk [8] and higher than for light-chain MGUS [13]. Among individuals with at least one high-risk feature by the 2/20/20 model [14], annual progression risk was ~9%. These findings support close clinical surveillance and further studies of risk stratification in this subgroup.

Strengths of our study include the population-based screened design of the iStopMM study, systematic bone marrow biopsies per protocol, and complete FLC analysis for all screened participants. Furthermore, state-of-the-art evaluations with MALDI-TOF-MS and NGF support our observations. Our findings were confirmed in a large population-based Danish cohort and describe the risk of progression.

In conclusion, light-chain SMM defined by abnormal FLC analysis, no detectable immunoglobulin heavy chain by SPEP or immunofixation, and BMPC 10–59% without myeloma-defining events identifies individuals with an annual progression risk of ~6%. This definition may improve early identification and monitoring strategies.

## Supplementary information

Supplementary

## Data Availability

De-identified data from iStopMM are available from the corresponding author upon reasonable request, subject to approval by the iStopMM investigators and the Icelandic National Bioethics Committee. Data from the DALY-CARE data resource can be accessed on a collaborative basis within the restrictions from the Danish National Ethics Committee. R codes are available upon request from corresponding author.
