# Supplementary material for: Redefining light-chain smoldering multiple myeloma: prevalence and progression risk
Source: Leukemia. 2026 Jul 6;40(9):2043–7. doi: 10.1038/s41375-026-03028-8 (PMC13506342; doi:10.1038/s41375-026-03028-8)
Supplement: Supplementary file 1 [file 41375_2026_3028_MOESM1_ESM.docx]

**Supplementary material**

**Supplementary Methods**

**Summary of the iStopMM study**

The iStopMM study is a prospective nationwide screening study for MGUS and randomized clinical trial of follow-up strategies (1). All residents of Iceland born before 1976 were offered screening for MM precursors with SPEP and FLC assay. Participants with a detectable M protein on SPEP or an abnormal FLC analysis defined as kappa/lambda light chain ratio <0.26 or >1.65 (or <0.37 or >3.1 for those with estimated glomerular filtration rate (eGFR) <60 mL/min/1.73m^2^) and an increase in the involved light chain entered a randomized controlled trial with three arms with the aim of evaluating the benefit and harms of screening for MM precursors. Individuals in arm 1 continued care in the Icelandic healthcare system as though they were never screened and were not informed of their screening results. Arms 2 and 3 were evaluated and followed at the study clinic with arm 2 receiving care according to current guidelines (plain skeletal surveys and bone marrow sampling for those with non-low risk MGUS or when clinically indicated, bone marrow sampling was omitted for most with light-chain MGUS). In arm 3 bone marrow sampling and whole-body low-dose computerized tomography (WBLDCT) were offered to all participants when feasible. UPEP was offered to all participants with >10% BMPC and negative SPEP, when possible.

Matrix-assisted laser desorption/ionization time-of-flight mass spectrometry (MALDI-TOF MS, (EXENT Immunoglobulin isotypes (GAM) assay, The Binding Site, part of Thermo Fisher Scientific) was performed on blood samples taken at light-chain SMM diagnosis. MS with FLC beads was performed in cases where the initial EXENT MS was negative(2). The EuroFlow next-generation flow (NGF)(3) method for minimal residual disease detection in MM (MM-MRD) was used to detect and quantify phenotypically aberrant BMPC.

The iStopMM study was approved by the National Bioethics Committee (NBC 16–022-04-26-2016) and the Icelandic Data Protection Authority and written consent was obtained from all participants in the study.

**The DALY-CARE cohort**

*The data origin*

The DALY-CARE data resource (4) contains data on all Danish adults registered with a lymphoid-linage cancer and lymphoid cancer precursors from 2002 to 2025. The data are collected from 10 different Danish registries and linked using the civil registration number. This includes national registries from the Danish Health Data Authority (including the Danish National Patient Registry (DNPR) (5,6) and the Danish National Pathology Registry), the Danish Multiple Myeloma Database (DaMyDa) (7) as well as electronic health record (EHR) data from Eastern Denmark.

Treatment data were derived from both the DPNR (5,6), EHR, the Danish National Prescription Registry, and DaMyDa (7). Importantly, DaMyDa captures ~100% of all registered MM diagnoses in Denmark from 2005, includes treatment data, and is expected to capture nearly all progressions (7). Any treatment not registered in DaMyDa (incl. for AL amyloidosis), we expect to detect in the remaining three registries. We extracted specific treatments (listed in Supplementary Table 1) as well as any anti-neoplastic treatments registered to a MM or AL amyloidosis diagnosis.

The DALY-CARE protocol was approved by the Danish Health Data Authority and National Ethics Committee (approvals P-2020-561 and 1804410, respectively). Analyses were performed in R software and the code is available upon request.

*Cohort construction*

Since SMM is formally registered as MM in the Internation Classification of Disease - Version 10 (ICD10) coding system, we defined SMM as individuals with a MM (ICD10 code: DC900) diagnosis who did not die or receive anti-myeloma treatment within 90 days of diagnosis. This cut-off has been used in previous studies on SMM (8) and aligns with our clinical experience.

FLC values were extracted from the National Laboratory Database (LABKA) and individuals without FLC values available were excluded from the study cohort. Bone marrow biopsy results were derived from DaMyDa (time of diagnosis) or the nation-wide pathology database (<90 days from diagnosis). Results from the latter were manually reviewed by a medical doctor (CVM). Individuals with <10% or >59% clonal plasma cells or missing bone marrow biopsy were not included (Supplementary Figure 2).

EHR were available from 46 individuals in the cohort and for 45 cases (98%) SMM diagnosis was confirmed by text search and one case (2%) remained undetermined due to insufficient documentation in the EHR.

Light-chain SMM was defined as SMM individuals without a detected intact heavy-chain M protein at any timepoint and abnormal values and ratio of FLC at diagnosis. For samples from laboratories using the FreeLite® assay, FLC ratio was considered abnormal if outside the iStopMM reference ranges (Supplementary Table 2)(9,10). For samples from laboratories using the N-Latex® assay, the FLC ratio was considered abnormal if outside the reference range of 0.31 to 1.56 regardless of renal function. Individuals were excluded if involved/uninvolved FLC ratio was ≥100 or if diagnosed with AL amyloidosis, solitary plasmacytoma, or plasma cell leukemia prior to or within 90 days light-chain SMM diagnosis as these diagnoses exclude the SMM diagnosis (Supplementary Figure 2).

For individuals in DALY-CARE, laboratory results, diagnoses, and text from electronic health records (EHR) were extracted from 90 days prior to registered treatment date to assess treatment indication. Treatment indications from the EHR were based on the clinicians’ descriptions.

**References**

1.	Rögnvaldsson S, Love TJ, Thorsteinsdottir S, Reed ER, Óskarsson JÞ, Pétursdóttir Í, et al. Iceland screens, treats, or prevents multiple myeloma (iStopMM): a population-based screening study for monoclonal gammopathy of undetermined significance and randomized controlled trial of follow-up strategies. Blood Cancer J. 2021 May 17;11(5):94. doi:10.1038/s41408-021-00480-w PubMed PMID: 34001889.

2.	Barnidge D, Troske D, North S, Wallis G, Perkins M, Harding S. Endogenous monoclonal immunoglobulins analyzed using the EXENT® solution and LC-MS. Journal of Mass Spectrometry and Advances in the Clinical Lab. 2024 Apr;32:31–40. doi:10.1016/j.jmsacl.2024.02.002

3.	Flores-Montero J, Sanoja-Flores L, Paiva B, Puig N, García-Sánchez O, Böttcher S, et al. Next Generation Flow for highly sensitive and standardized detection of minimal residual disease in multiple myeloma. Leukemia. 2017 Oct 20;31(10):2094–103. doi:10.1038/leu.2017.29

4.	Brieghel CWMFC et al. The Danish Lymphoid Cancer Research (DALY-CARE) Data Resource: The Basis for Developing Data-Driven Hematology. Clin Epidemiol. 2025.

5.	Schmidt M, Schmidt SAJ, Sandegaard JL, Ehrenstein V, Pedersen L, Sørensen HT. The Danish National Patient Registry: a review of content, data quality, and research potential. Clin Epidemiol. 2015;7:449–90. doi:10.2147/CLEP.S91125 PubMed PMID: 26604824.

6.	Schmidt M, Schmidt SAJ, Sandegaard JL, Ehrenstein V, Pedersen L, Sørensen HT. The Danish National Patient Registry: a review of content, data quality, and research potential. Clin Epidemiol. 2015 Nov;449. doi:10.2147/CLEP.S91125

7.	Gimsing P, Holmstrøm M, Wirenfelt Klausen T, Frost Andersen N, Gregersen H, Pedersen RS, et al. The Danish National Multiple Myeloma Registry. Clin Epidemiol. 2016 Oct;Volume 8:583–7. doi:10.2147/CLEP.S99463

8.	Sørrig R, Klausen TW, Salomo M, Vangsted AJ, Østergaard B, Gregersen H, et al. Smoldering multiple myeloma risk factors for progression: a Danish population-based cohort study. Eur J Haematol. 2016 Sep;97(3):303–9. doi:10.1111/ejh.12728 PubMed PMID: 26710662.

9.	Long TE, Indridason OS, Palsson R, Rognvaldsson S, Love TJ, Thorsteinsdottir S, et al. Defining new reference intervals for serum free light chains in individuals with chronic kidney disease: Results of the iStopMM study. Blood Cancer J. 2022 Sep 14;12(9):133. doi:10.1038/s41408-022-00732-3

10.	Einarsson Long T, Rognvaldsson S, Thorsteinsdottir S, Sverrisdottir IS, Eythorsson E, Oskarsson JT, et al. New Definition of Light Chain Monoclonal Gammopathy of Undetermined Significance. JAMA Oncol. 2025 Jul 1;11(7):753–61. doi:10.1001/jamaoncol.2025.1285 PubMed PMID: 40440024.

**Supplementary Table 1: Anti-myeloma treatments included in the study**

| *The Danish National Prescription Registry* | L01AA03: Melphalan  L01AA01: Cyclophosphamide  L01DB01: Doxorubicin  L04AX02: Thalidomide  L04AX04: Lenalidomide  L04AX06: Pomalidomide  L01XG01: Bortezomib  L01XG02: Carfilzomib  L01XG03: Ixazomib  L01FC01: Daratumumab  L01FX08: Elotuzumab  L01XH03: Panobinostat A01AC02: Dexamethasone |
| --- | --- |
| *National patient registry – Hospital administered treatments* | BWHA154: Melphalan  BWHA105: Cyclophosphamide  BWHA102: Doxorubicin  BWHB81: Thalidomide  BWHB82: Lenalidomide  BWHB86: Pomalidomide  BWHA402: Bortezomib  BWHA432: Carfilzomib  BWHA439: Ixazomib  BOHJ19H8: Daratumumab  BOHJ19H9: Elotuzumab  BWHA445: Panobinostat MH02AB02: Dexamethasone  Or any cytostatic treatment (BWHA) attached to diagnosis of plasma cell disorder. |

**Supplementary Table 2**: iStopMM revised reference intervals for free light chains using the Freelite assay

| Reference intervals for free light chains for FreeLite | | | | | | | |
| --- | --- | --- | --- | --- | --- | --- | --- |
| Renal function* | Age |  | **Kappa (mg/L)** |  | **Lambda (mg/L)** |  | **FLC ratio** |
| **Normal**  (eGFR* $\geq$ 60) | <70 years |  | 6.3 – 39.0 |  | 5.9 – 36.7 |  | 0.44 – 2.16 |
|  | $\geq$70 years |  | 7.0 – 55.8 |  | 6.4 – 48.0 |  | 0.46 – 2.59 |
| **Impaired** |  |  |  |  |  |  |  |
| eGFR 45 – 59 | |  | 7.8 – 83.6 |  | 7.3 – 65.1 |  | 0.46 – 2.62 |
| eGFR 30 – 44 | |  | 8.8 – 103.3 |  | 8.2 – 73.2 |  | 0.48 – 3.38 |
| eGFR < 30 | |  | 11.7 – 265.1 |  | 12.6 – 150.9 |  | 0.54 – 3.30 |

**Supplementary Figure 1: The iStopMM study design**. A total of 80 759 individuals provided informed consent to participate in iStopMM, and 75 422 underwent screening between 2016 and 2020. Of those screened, 3 788 had an abnormal test result and were randomized into one of three study arms. Participants in arm 1 continued routine care within the Icelandic healthcare system and were not informed of their screening results. Participants in arms 2 and 3 were evaluated at the study clinic: arm 2 followed standard guidelines, with skeletal surveys and bone marrow sampling performed based on risk stratification (bone marrow sampling was omitted in most cases of light-chain MGUS), whereas arm 3 offered bone marrow sampling and whole-body low-dose CT to all participants when feasible. Based on the initial evaluation, 193 individuals were diagnosed with SMM including 23 who met criteria for light-chain SMM.BM, bone marrow; FLC, free light chain; LC-SMM, light-chain smoldering multiple myeloma; MGUS, monoclonal gammopathy of undetermined significance; SPEP, serum protein electrophoresis.

**Supplementary Figure 2**: STROBE diagram of DALY-CARE cohort contruction
